# Robustifying Experimental Tracer Design for^13^C-Metabolic Flux Analysis

**DOI:** 10.3389/fbioe.2021.685323

**Published:** 2021-06-22

**Authors:** Martin Beyß, Victor D. Parra-Peña, Howard Ramirez-Malule, Katharina Nöh

**Affiliations:** ^1^Institute of Bio- and Geosciences, IBG-1: Biotechnology, Forschungszentrum Jülich GmbH, Jülich, Germany; ^2^Computational Systems Biotechnology (AVT.CSB), RWTH Aachen University, Aachen, Germany; ^3^Escuela de Ingeniería Química, Universidad del Valle, Cali, Colombia

**Keywords:** ^13^C-metabolic flux analysis, experimental design, isotope labeling experiments, *Streptomyces clavuligerus*, robustification, information criteria

## Abstract

^13^C metabolic flux analysis (MFA) has become an indispensable tool to measure metabolic reaction rates (fluxes) in living organisms, having an increasingly diverse range of applications. Here, the choice of the^13^C labeled tracer composition makes the difference between an information-rich experiment and an experiment with only limited insights. To improve the chances for an informative labeling experiment, optimal experimental design approaches have been devised for^13^C-MFA, all relying on some a priori knowledge about the actual fluxes. If such prior knowledge is unavailable, e.g., for research organisms and producer strains, existing methods are left with a chicken-and-egg problem. In this work, we present a general computational method, termed robustified experimental design (R-ED), to guide the decision making about suitable tracer choices when prior knowledge about the fluxes is lacking. Instead of focusing on one mixture, optimal for specific flux values, we pursue a sampling based approach and introduce a new design criterion, which characterizes the extent to which mixtures are informative in view of all possible flux values. The R-ED workflow enables the exploration of suitable tracer mixtures and provides full flexibility to trade off information and cost metrics. The potential of the R-ED workflow is showcased by applying the approach to the industrially relevant antibiotic producer *Streptomyces clavuligerus*, where we suggest informative, yet economic labeling strategies.

## 1. Introduction

Industrial biotechnology uses microorganisms as bio-factories for the production of a wide range of valuable compounds, ranging from food and feed additives, over biofuels to pharmaceuticals. The transition to a bio-based economy needs to establish sustainable yet economic processes with chassis organisms that achieve maximal yield and productivity (Lee et al., 2012; Dai and Nielsen, 2015). To achieve this chief goal, metabolic engineering strategies seek to orchestrate the material flows to transform resources efficiently to the target products (Stephanopoulos et al., 1998). In metabolic engineering, “omics” technologies nowadays have a fixed place, shedding light on the different molecular aspects of the cellular network. With the genome constituting the cellular inventory, its capacity (transcriptomics, proteomics) together with the thermodynamic driving force (metabolomics) determine the metabolic phenotype (fluxome). Hence, the knowledge of the fluxome, i.e., the set of all metabolic reaction rates (fluxes) in a living cell, plays a primary role in explaining how the phenotype and biological function actually manifests in a cell (Nielsen, 2003; Sauer, 2006).

The leading method for the accurate quantification of *in vivo* fluxes is^13^C metabolic flux analysis (^13^C-MFA) (Wiechert, 2001).^13^C-MFA uses mathematical modeling to infer the fluxes from data gathered in isotope labeling experiments (ILEs), i.e., external rate measurements and the labeling signatures of metabolic intermediates, to produce a so-called metabolic flux map. Any^13^C-MFA study starts with the question of how the ILE should be configured so that the risk of producing non-informative data, in terms of the desired fluxes, is minimized. This explains, why *in silico* experimental design (ED) has a longstanding tradition in^13^C-MFA and has become a basic module in the^13^C-MFA workflow (Möllney et al., 1999; Wiechert et al., 2001; Antoniewicz, 2013). In practice, also resource considerations (in terms of workforce, time, and money) play a non-negligible role when configuring ILEs (Nöh et al., 2018). In particular, labeled substrates are a substantial cost factor. With rare exceptions (Rantanen et al., 2006; Nöh et al., 2018), ED efforts in^13^C-MFA have concentrated on the identification of the most informative tracers for single (Möllney et al., 1999; Nöh and Wiechert, 2006; Metallo et al., 2009) and multiple (Crown et al., 2016) ILEs. More recently, also tracer costs have been considered (Bouvin et al., 2015; Nöh et al., 2018).

Besides the budget, the decision about the ILE configuration rests on information metrics that estimate the expected information gain of the ILE. Several of these metrics have been proposed in the statistical literature (Pukelsheim, 1993), all relying on flux confidence intervals as determined by statistical techniques such as linearized statistics (Wiechert et al., 1997) or profile likelihoods (Antoniewicz et al., 2006). Either way, the tracer design depends on an informed guess of the true fluxes, which are, however, to be determined by the ILE. In practice, such knowledge is not always at hand in the ILE planning stage, e.g., in the case of new strains or unusual substrates. Clearly, when in such situation the mathematical model describing the input (substrate)—output (labeling) relation is highly non-linear in the flux values, any approach conditioned on one single flux guess risks rendering the design choice sub-optimal or even meaningless. This leaves the field with a chicken-and-egg dilemma.

In the situation of limited knowledge it is therefore warranted to robustify the tracer design by making it less sensitive to the assumed fluxes. One solution to this is to employ a multi-experiment design strategy, where a sequence of ILEs is planned using the acquired information from the previous experiments to design the next ILE, therewith consecutively narrowing down the flux ranges (Körkel et al., 2004). Unfortunately, this approach is often impractical for^13^C-MFA due to time and cost constraints. Another ED strategy to tackle the uncertainty in the fluxes is to cast the design task into a worst-case formulation (Pronzato and Walter, 1988; Asprey and Macchietto, 2002). Technically, such approaches yield bi-level optimization problems to minimize the maximal expected confidence region of the unknown fluxes, which are typically difficult to treat (Hettich and Kortanek, 1993), even when considering relaxed approximate formulations (Beyer and Sendhoff, 2007). More importantly, even though worst-case solutions deliver a guaranteed minimum of information under all possible circumstances, these designs may be far from being informative on average.

Here, we pursue a single-experiment approach that yields tracer designs, which are immunized against the uncertainty in the initial flux “guesstimates”. The methodology, called robustified ED (R-ED), relies on flux space sampling to compute design criteria for the whole range of possible fluxes. The sampled EDs are then screened for best compromise solutions, where the “best” design can be tailored a posteriori to account for new circumstances or policy changes. Hence, instead of computing a single best tracer mixture, an exploration-based decision process is followed that allows to take practical constraints, such as commercially available amounts of labeled species or mixture complexity, into consideration once the set of EDs is available. This enables to keep the design strategy flexible, e.g., to switch between a design tailored to particular subsets of fluxes or a design which targets as many as possible fluxes simultaneously. To provide a generally applicable framework, the R-ED workflow relies on^13^C-MFA models specified in the universal model description language FluxML (Beyß et al., 2019). To facilitate easy adoption, the R-ED workflow is assembled from standard computational^13^C-MFA elements, being available for example in the high-performance simulation software suite 13CFLUX2 (Weitzel et al., 2013). We showcase our approach with *Streptomyces clavuligerus*, a clavulanic acid (CA) producing bacterium of interest for pharmaceutical industries. For this organism, metabolic models have been reconstructed, but a deep characterization of the fluxome is yet lacking.

## 2. Materials and Methods

### 2.1. Modeling, Evaluation, and Analysis Frameworks

The^13^C-MFA network model, including flux constraints, extracellular rate and labeling measurements was specified visually, following the workflow described in Nöh et al. (2015), using the network editor and visualization software Omix (Omix Visualization, Lennestadt, Germany, Droste et al., 2011). The network model was formulated in the universal flux modeling language FluxML (Beyß et al., 2019). The FluxML model file served as input for the high-performance^13^C-MFA simulation suite 13CFLUX2 (Weitzel et al., 2013), with which all simulation tasks and statistical analyses were performed. Simulation results are stored in binary HDF5 (Hierarchical Data Format version 5) files (The HDF Group, 2021). For intermediate evaluation and processing tasks, such as the diagnosis of flux (non-)identifiability and compiling the results, custom Python and Matlab (The MathWorks, Inc., Natick MA, USA) scripts were developed that enable the fully automated execution of the evaluation. All scripts are included in the Supplementary Information to guarantee reproducibility of the data analyses and to allow for replication experiments. The complete execution pipeline is documented in the Supplementary Information S1 (Section S2).

### 2.2. Construction of a^13^C-MFA Model for *S. clavuligerus*

**Figure 1:**
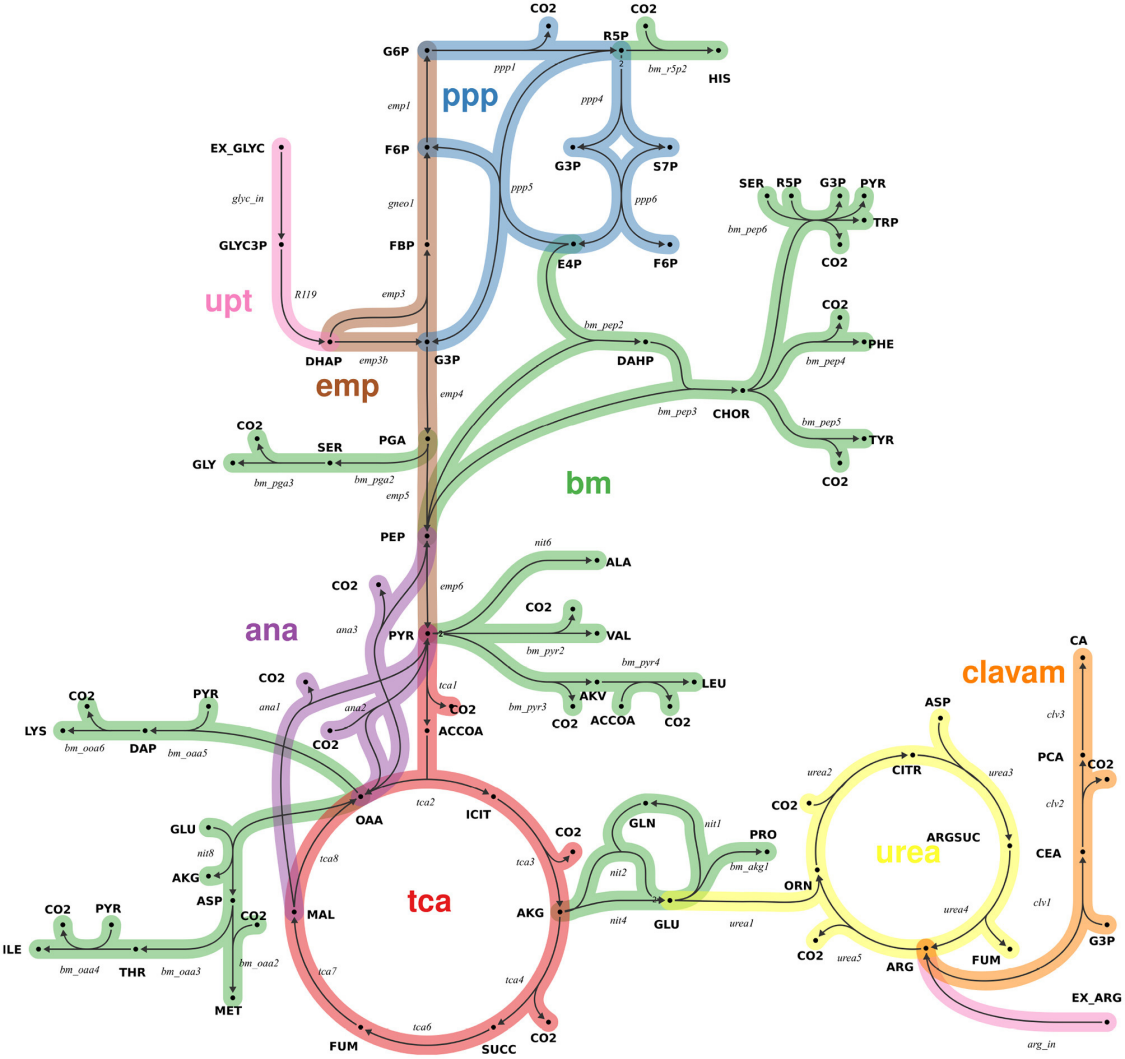
Metabolic network model of the central carbon metabolism of *S. clavuligerus* (simplified view). The model comprises 48 intracellular metabolites (bold) and 89 reactions (italic), grouped into 8 pathways (color-coded): glycolysis (emp), pentose phosphate pathway (ppp), tricarboxylic acid cycle (tca), anaplerosis (ana), urea cycle (urea), clavam biosynthesis pathway (clavam), uptake (upt) and lumped biosynthesis reaction for biomass (bm). The full model specification in FluxML format is found in Supplementary Data S2.

A^13^C-MFA network model of the core central metabolism of *S. clavuligerus* was formulated based on previous work (Medema et al., 2010; Ramirez-Malule et al., 2016, 2018; Gómez-Ríos et al., 2020). Reactions of CA biosynthesis via the clavam pathway were added (Ramirez-Malule et al., 2018). A visual overview of main reaction pathways is provided in Figure 1. All main metabolic pathways of central carbon metabolism were included in the model, including glycolysis (emp), pentose phosphate pathway (ppp), tricarboxylic acid (tca) cycle, anaplerotic reactions, urea cycle, clavam pathway, as well as lumped amino acid biosynthesis pathways. Cellular growth was modeled based on the biomass composition measured for *Streptomyces coelicolor* (Borodina et al., 2005), due to strong evidence that the genome core between the two *Streptomyces* strains is highly conserved (Alam et al., 2011). Here, for each biomass component one efflux was formulated, with a flux value given by the respective biomass contribution multiplied by the dilution rate. Reaction bidirectionalities were formulated according to Bouvin et al. (2015). For all intracellular reactions, carbon atom transitions were formulated. The^13^C-MFA model consists of 48 intracellular metabolites and 89 reactions (74 uni- and 15 bidirectional), has two uptake reactions [for glycerol (GLYC) and arginine (ARG)] and has 22 independent (free) flux parameters (7 net and 15 exchange fluxes) (Wiechert and de Graaf, 1996). All net fluxes are constrained by a lower and upper bound of ± 100% of the GLYC uptake, respectively. The exchange fluxes of all bidirectional reaction steps were constrained by an upper bound of 200% of the GLYC uptake. The complete model, specified in the universal flux modeling language FluxML, is provided in the Supplementary Data S2.

### 2.3. Experimental Design Setting for the^13^C-MFA Study

For our study, we conceived an experimental setup for *S. clavuligerus* that is relevant to characterize metabolic fluxes under CA production conditions (Sánchez et al., 2015; Ramirez-Malule et al., 2018).

*Isotope labeling experiment: S. clavuligerus* is cultivated under chemostat conditions at a dilution rate of 0.03 h^-1^ (Bushell et al., 2006). A growth medium is considered that contains GLYC and ARG as sole carbon sources in concentrations of 20 and 0.17 g/L, respectively, which was found beneficial for CA production (Ser et al., 2016). Therefore, GLYC (C-3) and ARG (C-5), both feeding into clavam biosynthesis, qualify as tracers. All commercially available isotopic species are considered in the ED study. These are [U-^13^C_3_]-, [1,3-^13^C_2_]-, [2-^13^C_1_]-, and [^12^C]-GLYC, and [U-^13^C_6_]-, [6-^13^C_1_]-, and [^12^C]-ARG. For a purity of 99 atom%, the costs of these tracers vary between 0.36 $/g ([^12^C]-GLYC) and 3,449 $/g ([U-^13^C_6_]-ARG). The ILE is performed in a bioreactor with a working volume of 250 ml. To achieve isotopic stationarity of the label in protein-bound amino acids, samples are withdrawn after 5 bioreactor residence times (approximately 20 cell divisions) (Wiechert and Nöh, 2005). Full specification of tracers used in this study is available in the Supplementary Information S1 (Table S1).

*Measurements:* Extracellular rate measurements for the uptake of ARG, CA production and CO 2 secretion are assumed to be available. Standard deviations of these rates are formulated based on experience (5% for ARG and CA, 20% for CO2). The specific GLYC uptake rate of 4.9 mmol GLYC/gCDW/h (*glyc_in*) is scaled to a value of 100 and all fluxes are related to this rate. The ARG uptake rate (*arg_in*) is constrained to values below 3% of the *glyc_in*, as estimated from the demand for CA production (Bushell et al., 2006).

For measuring isotopic labeling patterns of proteinogenic amino acids, gas chromatography–mass spectrometry (GC–MS) was selected as analytical tool, being the predominantly used analytical device in^13^C-MFA. Mass isotopomer distributions of 16 commonly detected amino acids comprising 41 measurement groups (167 independent measurements in total) are assumed (Supplementary Information S1, Table S2). For the prediction of the expected measurement error, the following linear error model was employed:

(1) $$\begin{array}{r} \sigma \left({\text{y}}^{meas}\right)=4.120\;10^{-2}\cdot {\text{y}}^{meas}+6.655\;10^{-3} \end{array}$$

as derived from a previous literature survey (Nöh et al., 2018). The complete measurement configuration model is found in the FluxML file (Supplementary Data S2).

### 2.4. Traditional Optimal ED Workflow for^13^C-MFA

To introduce the formal notation used throughout and for convenience of the reader, we recapitulate the essentials of the traditional workflow for the optimal design of isotopic tracer experiments. At metabolic steady-state, the intracellular flux vector **v** of a given metabolic reaction network, obeys a linear stoichiometric system:

(2) $$\begin{array}{r} \text{S}\cdot \text{v}=0,\;\text{C}\cdot \text{v}\leq \text{c} \end{array}$$

with the *m* × *n* dimensional stoichiometric matrix **S** (with *m* < *n*), and additional linear flux constraints to exclude physiologically meaningless states, to set reaction directions, or to equate fluxes of scrambling reactions (Wiechert and de Graaf, 1997). Therewith, the system in Equation (2) defines the flux solution space **V**, a convex polytope. A basis of **V** is given by the independent (or free) fluxes **v**^*free*^ ∈ ℝ^*n*−*rank*(**S**)^ (Wiechert and de Graaf, 1996). Notice that while the number of independent fluxes is unique, the choice of the free flux set constituting the vector **v**^*free*^ is non-unique. Since the knowledge of **v**^*free*^ is sufficient to determine the complete flux vector **v**, the attribute “free” is omitted for brevity, unless otherwise noted.

The aim of^13^C-MFA is to determine the fluxes that best explain a set of isotopic steady-sate labeling measurements **y**. To this end, a^13^C-MFA model is formulated with which the labeling measurements **y** are calculated, given some feasible fluxes and substrate mixture **x**^*inp*^, which is a cocktail composed by available substrate species:

(3) $$\begin{array}{r} \text{y}=f\left(\text{v},{\text{x}}^{inp}\right)+\epsilon ,\text{with}\epsilon \sim N\left(0,\Sigma \right),\text{v}\in \text{V},{\text{x}}^{inp}\in {\text{X}}^{inp} \end{array}$$

The non-linear function ***f*** follows from mass balancing of the atom transition model (Wiechert et al., 1999). Here, the measurement errors **ϵ** are assumed to be independent and normally distributed with zero mean and measurement covariance matrix **Σ**.

The information about how precise the fluxes are expected to be, given the measurements is captured in the approximate flux covariance matrix **Cov**, which is derived from the Fisher Information matrix **FIM**. The **FIM** relies on the first order sensitivities (i.e., the Jacobian) of the labeling system (3) at some reference flux vector **v**^⋆^:

(4) $$FIM\left(v^{⋆},x^{inp},\Sigma \right)={\frac{\partial y}{\partial v}|}_{v^{⋆}}^{T}\cdot \Sigma^{-1}\cdot {\frac{\partial y}{\partial v}|}_{v^{⋆}}$$

Hence, the **FIM**, and therefore the estimated flux covariance matrix **Cov**, can be influenced by deliberate choice of the design variables: the composition and errors of the measurements, **y** and **Σ** (both considered fixed in this study), as well as the input substrate composition **x**^*inp*^.

With Equation (4) the cyclic problem of non-linear ED becomes apparent: the **FIM** relies on the assumption that **v**^⋆^ is a good approximation of the true fluxes, which, however, we wish to determine with the ILE. Depending on the non-linearity of the^13^C-MFA model (3), evaluating Equation (4) at some other flux vector may yield a different **FIM** and, consequently, different approximate flux (co)variances:

(5) $$\begin{array}{r} \text{C}\text{o}\text{v}\left({\text{v}}^{⋆},{\text{x}}^{inp},\Sigma \right)={\text{F}\text{I}\text{M}}^{-1}\left({\text{v}}^{⋆},{\text{x}}^{inp},\Sigma \right) \end{array}$$

This procedure comes with an additional caveat: to ensure that a design is non-degenerate, the **FIM** is required to be invertible. Technically, invertibility is enforced by imposing lower and upper thresholds for the singular values and the condition number of the **FIM** (Golub and van Loan, 1996). Such thresholds are then met by removing statistically non-identifiable fluxes by fixing their value to the respective reference value in **v**^⋆^ (Wiechert and de Graaf, 1997; Isermann and Wiechert, 2003). In reality this means that the inversion leads to a dimension reduction of the free flux space, which ties the design even tighter to the flux guesstimate. Formally, we call the selection of the remaining *n*_*act*_ identifiable free fluxes *active fluxes*, and denote them by **v**_*act*_. The removal of statistically non-identifiable fluxes is not necessarily unique, implying that any criterion relying on the covariance matrix depends on the choice made. For simplicity, out of all possible active flux sets, the one with the overall minimal covariances is selected. Consequently, the covariance matrix depends on the choice of **v**_*act*_. To not overload notation, we omit this dependency for brevity.

For the purpose of ED, the information about the expected flux precision is condensed to a single value, giving raise to a criterion which is maximized by varying the tracer mixture. For this, multiple “alphabetical” information criteria have been proposed (Pukelsheim, 1993). The most prominent among these is the D-criterion, which measures the volume of the expected flux confidence ellipsoid in the (dimension-reduced) flux space:

(6) $$\begin{array}{r} \Phi_{D,n_{act}}\left({\text{x}}^{inp},{\text{v}}^{⋆}\right)=\sqrt[{2n_{act}}]{\frac{\det\left(\text{C}\text{o}\text{v}\left({\text{v}}^{⋆},{\text{x}}^{inp,ref},\Sigma \right)\right)}{\det\left(\text{C}\text{o}\text{v}\left({\text{v}}^{⋆},{\text{x}}^{inp},\Sigma \right)\right)}} \end{array}$$

Here, for the ease of interpretability, the D-criterion values are related to some predefined reference ILE. In this way, values larger (smaller) than 1 indicate that the design is improved (worse) as compared to the reference tracer.

In optimal ED (O-ED) designs with a high number of identifiable fluxes are preferred (requiring a minimal number of flux fixations). This implies that optimal designs are often conditioned on the highest possible *n*_*act*_, where *n*_*act*_ is usually found by an iterative procedure. Nonetheless, also partial designs, targeting only a sub-set of the fluxes, may be desired (Möllney et al., 1999). We subsume all cases under the same notation, indicated by the effective dimensionality of the criterion value:

(7) $$\begin{array}{r} \underset{{\text{x}}^{inp}\in {\text{X}}^{inp}}{\max}\Phi_{D,n_{act}}\left({\text{x}}^{inp},{\text{v}}^{⋆}\right) \end{array}$$

It should be remarked that in Equation (7) instead of the D-criterion any other information metric may be used.

**Figure 2:**
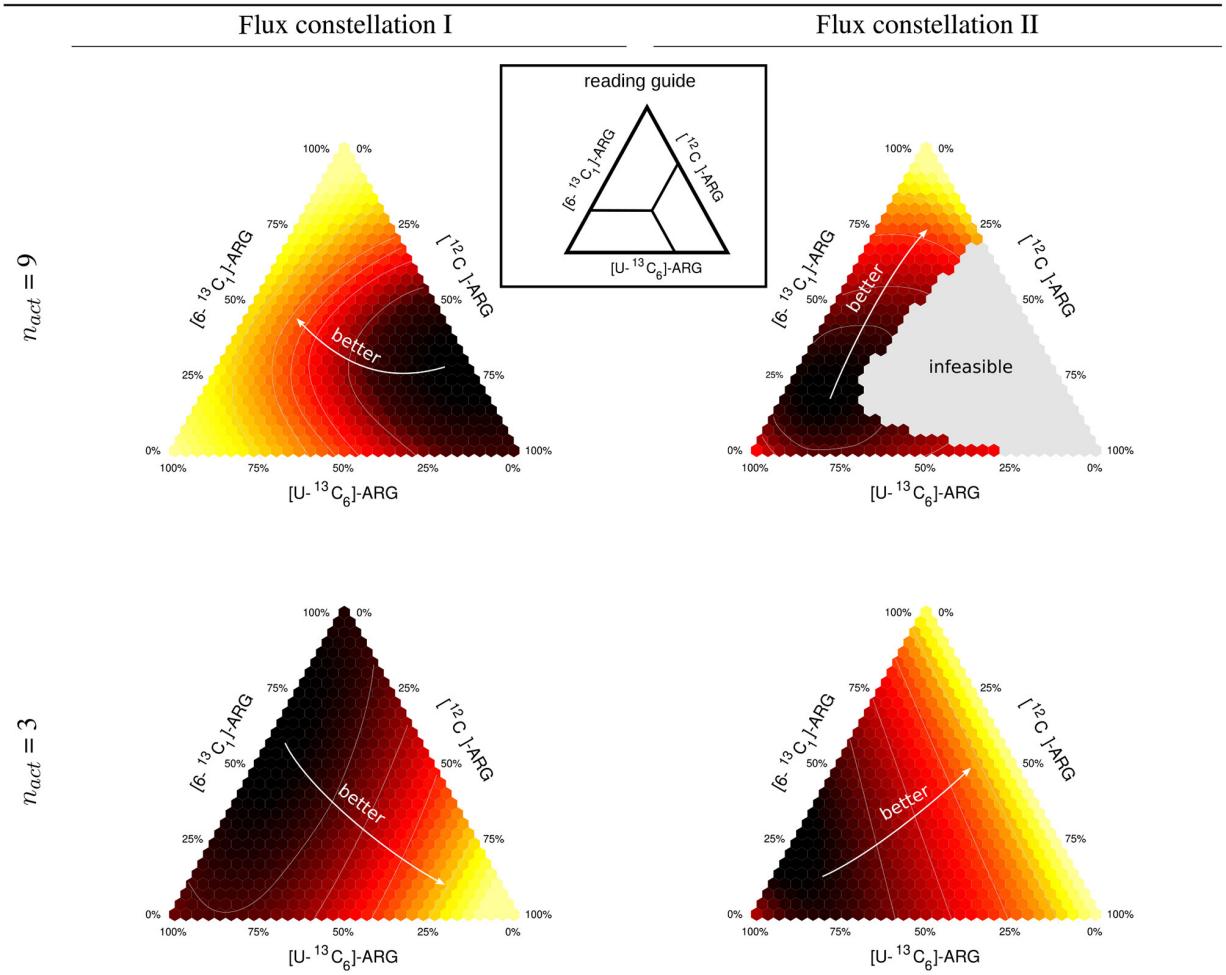
Expected information for two different flux constellations. Ternary triangles for mixtures of [^12^C], [U-^13^C_6_], and [6-^13^C_1_] tracer species of ARG and [2-^13^C_1_]-GLYC. The triangles are calculated for two different flux constellations (I and II) differing, inter alia, in the clavam pathway flux (see also Supplementary Information S1, Table S3). The upper row gives results for a full design (9 active fluxes), the lower row for a dimension-reduced design (3 active fluxes).

In practice,^13^C-MFA tracer mixture compositions contain no more than a handful of species. Hence, Equation (7) is solved by grid searching. For a substrate with *s* different tracer species evaluated on an equally spaced grid with an interval distance of *d*, this amounts to

(8) $$\frac{1}{\left(s-1\right)!}\;\prod_{i=0}^{s-2}\left(\frac{1}{d}+1+i\right)$$

design combinations to be tested and collected. Finally, these D-criterion values Φ_*D*,__*n*_*act*__ are visualized in mixture triangles from which the optimal tracer combination is read off (cf. Figure 2). The computational O-ED workflow is summarized in Algorithm 1.

**Algorithm 1:**
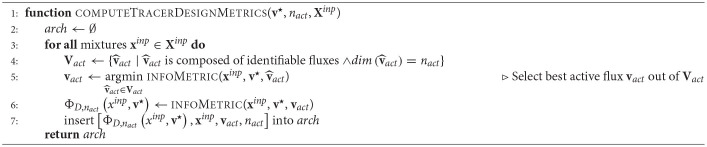
Traditional optimal tracer design.

To summarize, the assumption that **v**^⋆^ is a good estimate of the true fluxes, conditions the traditional O-ED to the flux guesstimate. In addition, covariance-based criteria relying on an inversion of the **FIM** may encounter the need for a dimensional reduction of the ED, which also ties the design to the flux guesstimate.

## 3. Results and Discussion

### 3.1. When Traditional ED Is of Limited Utility: An Illustrative Example

In this work, we take the industrially relevant non-model organism *S. clavuligerus* as an example. The Gram-positive bacterium is a natural producer of CA, a potent inhibitor of β-lactamase enzymes secreted by bacteria as a defense mechanism against β-lactam antibiotics (Brown et al., 1976; Ramirez-Malule, 2018; Ramirez-Malule et al., 2018; López-Agudelo et al., 2021). *S. clavuligerus* grows on GLYC as main carbon source, while amino acid supplementation, e.g., ARG, improves CA titer. GLYC is converted to glyceraldehyde-3-phosphate and then incorporated to the β-lactam ring of the CA molecule via the clavam pathway, whereas ARG provides carbon for the remaining part of the metabolite. Precise quantitative information about the intracellular fluxes from these substrates toward the clavam pathway is lacking so far. We take the experimental scenario of CA production with *S. clavuligerus* as an example to showcase the shortcomings of the traditional O-ED workflow (Algorithm 1).

We selected two different flux constellations from the space of stoichiometrically feasible ones. Flux constellation I is characterized by a low flux through the urea cycle and a high CA flux, whereas flux constellation II has a low flux through the clavam pathway, mimicking low CA production, and consequently a high CO 2 secretion and metabolically active urea cycle. The values for both flux constellations are available in Supplementary Information S1 (Table S3).

For a [2-^13^C_1_]-GLYC tracer we explored the D-criterion landscape for mixtures of three ARG species, namely [^12^C]-, [U-^13^C_6_]-, and [6-^13^C_1_]-ARG. The results in terms of Φ_*D*_ are represented by ternary plots shown in Figure 2. First, for 9 active fluxes (the maximal number of identifiable fluxes out of 22 free fluxes with the reference mixture), we found mixtures that are informative for both constellations, namely mixtures that are dominated by [6-^13^C_1_]-ARG, and unfavorable mixtures, primarily mixtures with a high proportion of [^12^C]-ARG. In particular, the information-less mixtures migrate from 70% [^12^C]-ARG and 5% [U-^13^C_6_]-ARG for flux constellation I to 15% [^12^C]-ARG and 75% [U-^13^C_6_]-ARG for flux constellation II, while the proportion of [6-^13^C_1_]-ARG remains similar. Consequently, whereas for constellation I, 10% [6-^13^C_1_]-ARG and 90% [U-^13^C_6_]-ARG is a highly informative mixture, it performs poorly for constellation II. Strikingly, constellation II shows a large gray region in the mixing triangle, representing tracer compositions leading to a singular **FIM**, meaning that these mixtures are unable to identify some of the 9 active fluxes.

When reducing *n*_*act*_ to 3, a minimum of three fluxes can be identified by all tested mixtures. However, compared to *n*_*act*_ = 9, the design results, captured by the mixing triangles, change drastically. This could be expected, since full and dimension-reduced designs have a different set of target fluxes. The previously information-less mixtures with low [U-^13^C_6_]-ARG content are among the best performers. Also in this case, the information landscapes of the two flux constellations have different characteristics. Mixtures with low [6-^13^C_1_]- and [U-^13^C_6_]-ARG portions are clearly favored by constellation I, whereas for constellation II the proportions of [6-^13^C_1_]- and [^12^C]-ARG tracers do not matter as long as the contribution of [U-^13^C_6_]-ARG remains small.

In summary, the optimal mixture can be quite different for different feasible flux constellations. As such, this is not problematic, as long as the flux (co)variances remain coherent over the flux space. In the example, this coherence is, however, broken as indicated by the large gray area for the full design (*n*_*act*_ = 9). This motivates the introduction of a criterion that favors designs being informative on average, in view of the whole relevant flux space. Secondly, it appears beneficial to delay the design decision on the modeling choice of *n*_*act*_ to the post-processing phase, rather than conditioning the design decision on it.

### 3.2. Robustified Experimental Tracer Design Workflow R-ED

In a situation, where precise knowledge about the true flux distribution is lacking, tracer designs that are informative for a wide range of possible flux values (i.e., robustified designs) are preferable over those designs that are confined to a specific flux setting (optimal designs). To make the design choice less vulnerable to assumptions about flux values, we need to account for all possible flux values. When no prior knowledge on the *in vivo* fluxes is available, hence, the whole feasible flux space **V**, as defined by (2), is to be considered. This comes with the need of an update for the information criterion.

#### 3.2.1. Criteria for Robustified Experimental Tracer Design

To characterize the robustness of substrate mixtures with respect to the flux uncertainty, we introduce three design criteria, two targeting the information gain of the ILE and one cost-related criterion. Both information criteria rely on a sample of flux constellations that are representative for the whole feasible flux space **V**. Here, the population is derived by random sampling of *n*_*S*_ flux maps **v**_*i*_, drawn from the convex flux space **V**. The resulting set of sampled flux maps is denoted **V**_*n*_*S*__ = {**v**_*i*_}_*i* = 1, …, *n*_*S*__.

For characterizing the informativeness of the designs over a set of sampled flux maps, taking the 2-quantile (median, **Q**_2_) of the D-criterion values over the set is an apparent choice:

(9) $$\begin{array}{r} {\hat{\Phi }}_{D,n_{act}}\left({\text{x}}^{inp}\right)={\text{Q}}_{2}\left\{\Phi_{D,n_{act}}\left({\text{x}}^{inp},{\text{v}}_{i}\right),\forall {\text{v}}_{i}\in {\text{V}}_{n_{S}}\right\} \end{array}$$

The higher the median, ${\hat{\Phi }}_{D,n_{act}}$, the smaller are the typically expected flux variances, and hence the more favorable is the design on average. Here, as noted in section 2.4, other alphabetical criteria work equally well.

A second criterion for robustified tracer design, motivated in the illustrative example, is the proportion of the flux space for which the particular tracer choice achieves *n*_*act*_ identifiable fluxes. We call this criterion the *coverage* of the tracer design. Precisely, the proportion of flux map samples **v**_*i*_ out of **V**_*n*_*S*__, for which at least one statistically identifiable flux set is found, defines the coverage:

(10) $$\begin{array}{l} {\hat{\Phi }}_{Cover,n_{act}}\left(x^{inp}\right)=\\ \frac{card\left(\left\{v_{i}\in V_{n_{S}}|x^{inp}\text{ has }n_{act}\text{ identifiable fluxes}\right\}\right)}{n_{\text{​}S}} \end{array}$$

This means, the higher the coverage of a substrate mixture, the higher is the chance to identify *n*_*act*_ fluxes over the relevant flux space **V**. We express the coverage in percents, where 0% (100%) indicates that none (all) of the flux samples have *n*_*act*_ identifiable fluxes. Notice, that the coverage criterion does not make a statement about how well the fluxes are identified. Conversely, the ${\hat{\Phi }}_{D,n_{act}}$ values carry no information about how likely it is to identify *n*_*act*_ fluxes.

Finally, as a third criterion, we take costs into account. Here, we restrict cost considerations to tracer costs as we have shown before that these are the by far predominant cost drivers (Nöh et al., 2018). The cost of each ILE is calculated by multiplying the relative abundance of each tracer species with their required absolute amounts and their costs per gram:

(11) $$\begin{array}{r} \Phi_{\$}\left({\text{x}}^{inp}\right)={\text{c}}^{T}\cdot {\text{x}}^{inp} \end{array}$$

#### 3.2.2. R-ED Workflow

In R-ED, we are interested in ILEs which maximize both information criteria (9) and (10), while remaining economically feasible. As motivated in the illustrative example, the selection of the best tracer design involves to take trade-off decisions between the objectives. The optimal trade-offs between the objectives, the so-called Pareto optima, can be obtained by multi-objective optimization approaches (Nöh et al., 2018). In many practically relevant situations, besides inspecting the Pareto optima, it may also be worthwhile to consider non-Pareto design sets. For example, not all tracers may be available at the assumed price or a dual mixture composition may be favored over a mix of four tracers, although it is slightly less informative. We therefore opted for an exploratory approach, which enables the experimenter to study the relationship between the substrate and the information criteria, to eventually make an informed decision on a useful substrate mixture.

The R-ED algorithm proceeds in three phases (cf. Algorithm 2). Firstly, for a given^13^C-MFA model the feasible flux space is sampled uniformly providing *n*_*S*_ flux map samples **V**_*n*_*S*__ (sampleFluxSpace). In case that prior knowledge about the fluxes exists, e.g., in form of a joint confidence ellipsoid, non-uniform Gaussian sampling can be employed instead (Jadebeck et al., 2020).

**Algorithm 2:**
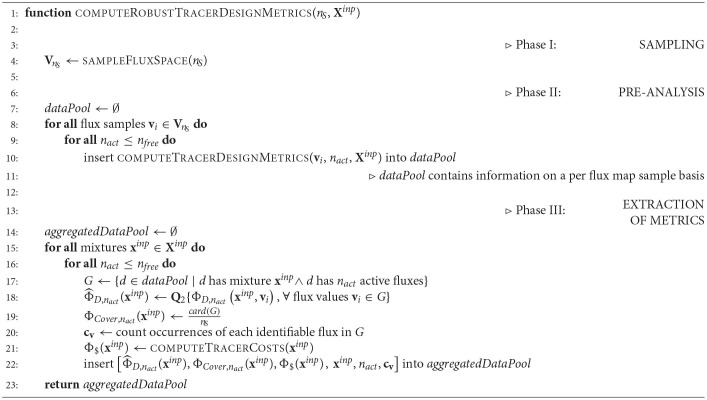
Robust tracer design.

In the second phase, the conventional O-ED module (Algorithm 1 computeTracerDesignMetrics) is applied to every flux map sample **v**_*i*_ (instead of just **v**^⋆^). For every *n*_*act*_ the information criterion Φ_*D*,__*n*_*act*__ and the selection of active fluxes *v*_*act*_ are collected in a *data pool*.

In the third phase, the three design metrics are extracted from the data pool. To this end, by iterating over all tracer combinations and identifiable (active) flux sets, the median-based D-criterion (9) and the coverage (10) are calculated. Furthermore tracer costs $\Phi_{\$}\left({\text{x}}^{inp}\right)$ are added (computeTracerCosts). Because we may be interested in particular fluxes to be identifiable, the number of times they contribute to the active flux sets is also derived. All these criteria are collected in the *aggregated data pool*, containing all relevant information for making design decisions. The aggregated data pool is then subjected to visual post-processing to identify a suitable experimental setting for the ILE as showcased below.

#### 3.2.3. Implementation of the R-ED Workflow

**Figure 3:**
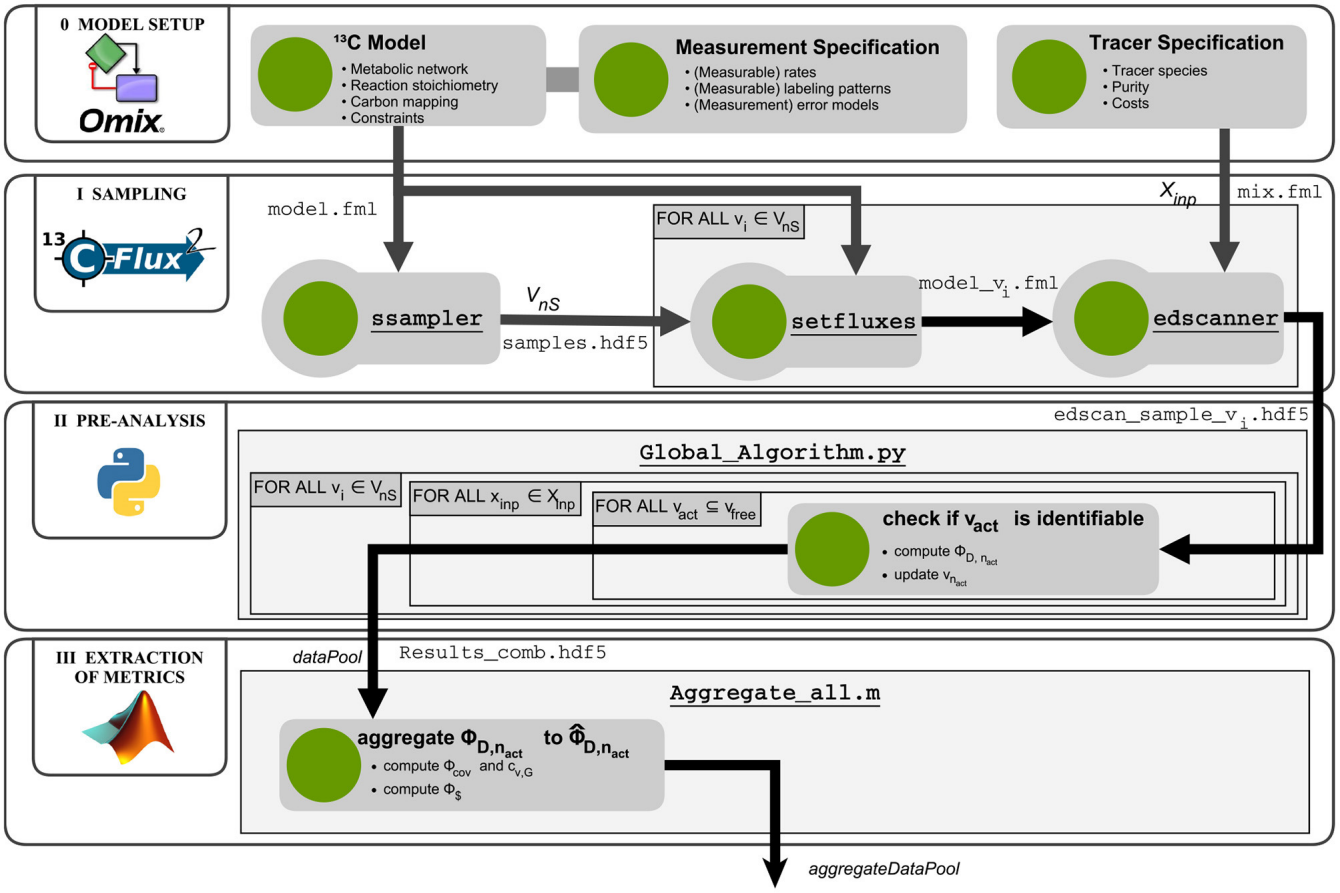
Computational workflow for robustified tracer design. The R-ED workflow consists of four main blocks: *Phase 0 - Model setup*: Assembly and configuration of the metabolic model with Omix; *Phase I - Sampling*: Determining a set of flux maps that are representative for the feasible flux space and computation of Jacobians of the labeling systems with 13CFLUX2; *Phase II - Pre-analysis*: Determination of active flux sets and calculation of design metrics for the flux map samples; *Phase III - Extraction*: Aggregating the results from different flux map samples to combined metrics. The aggregated data pool is then subjected to further analysis to support informed decision-making. Matlab and Python scripts are found in the Supplementary Data S3.

The R-ED workflow schematic is shown in Figure 3. As input, a valid^13^C-MFA model is required. One convenient way to set up a model is to employ the Omix software for visual network composition (for details see section 2.2). The model contains the information about reaction stoichiometry, carbon atom mappings, measurement configurations (external rates and label incorporation) together with their standard deviations, formulated in an FluxML model (model.fml). The FluxML model is validated against the FluxML language standard as defined in the W3C XML Schema www.13cflux.net/fluxml using the FluxML parser fmllint. Tracer species are specified, together with purity (atom%) and costs ($ per substrate feed), in separate tracer specification XML files (mix.fml). Exemplary model and tracer specification files are available in Supplementary Data S2.

In phase I, the flux space **V** is sampled uniformly. To this end, the ssampler tool of the high performance simulator 13CFLUX2 is called, which implements the Markov Chain Monte Carlo algorithm Hit-and-Run (Bélisle et al., 1993). Another alternative is to call the sampling suite HOPS (Jadebeck et al., 2020), a library which contains various efficient sampling algorithms specially tailored to polytope sampling. Sampling results are collected in a HDF5 file (samples.hdf5). Then, each individual sample **v**_*i*_ ∈ **V**_*n*_*S*__ is transferred to the model using the setfluxes command. With the resulting *n*_*S*_ FluxML models (model_v_i_.fml) and the tracer specification file mix.fml, the edscanner tool is called to generate the tracer mixture grid (${\text{X}}^{inp},\;card\left({\text{X}}^{inp}\right)=n_{T}$) of desired granularity. For all tracer combinations the first-order derivatives of the labeling states with respect to the fluxes (i.e., the Jacobians) are calculated with the 13CFLUX2 simulator. Per flux sample one Jacobian is generated and stored in an HDF5 file, resulting in *n*_*S*_ edscan_sample_v_i_.hdf5 files with *n*_*T*_ Jacobians each.

In phase II, information metrics are calculated for each flux sample – tracer mixture combination (Algorithm 1). The Fisher matrices are calculated from the measurement covariance matrices and the Jacobians generated in the previous phase, according to Equation (4). For computational efficiency, the calculation of the information metrics are performed in parallel with a Python script (Global_Algorithm.py. Results are collected in a data pool (Results_comb.hdf5).

In the final phase of the R-ED workflow, the data pool is further evaluated by the Matlab script Aggregate_all.m to aggregate the information criteria and to calculate the ILE costs. The aggregated data pool contains the information that can be interrogated from different angles to support decision-making about the ILE. Various strategies are possible, which are discussed in the next section. Further details about the workflow execution are provided in the Supplementary Information S1 (Section S2). Glueing and analysis scripts are available in Supplementary Data S3.

### 3.3. Applying the R-ED Workflow to *S. clavuligerus*

*Streptomyces* bacteria are known to be potent producers of a broad range of antibiotics (de Lima Procópio et al., 2012). The genus includes strains such as *S. griseus* for the production of streptomycin, *S. venezuelae* for chloramphenicol, or *S. fradiae* for fosfomycin, neomycin and tylosin (Okamoto et al., 1982; Demain and Sanchez, 2009). To increase treatment efficiency and to mitigate emerging bacterial resistances, combinations of antibiotics are administered. Here, one common strategy is to combine an antibiotic with a drug which inhibits the antibacterial defense system. For instance, resistance to β-lactam antibiotics is tackled by so-called β-lactamase inhibitors. One important inhibitor, which is used in conjunction with β-lactam antibiotics such as penicillin, is CA being produced by the filamentous bacterium *S. clavuligerus* (Brown et al., 1976). The combination of CA and amoxicillin is a well-established broad-spectrum antibacterial treatment (World Health Organization, 2019a,b), which was prescribed in 2018 almost 8 million times in the US alone (Kane, 2021). Although bioprocess development and genetic engineering successfully improved CA yields (Li and Townsend, 2006; Ünsaldı et al., 2017; Ramirez-Malule et al., 2018; Gómez-Ríos et al., 2019; López-Agudelo et al., 2021), rational metabolic engineering based on the precise knowledge of the fluxome of *S. clavuligerus* is expected to push the limits further. The knowledge about *in vivo* fluxes is, however, still very limited (Medema et al., 2010; Ramirez-Malule et al., 2018; Gómez-Ríos et al., 2020), which makes this system an ideal showcase for the proposed R-ED methodology.

For the production of CA with *S. clavuligerus* two carbon sources are essential, GLYC and ARG. For both a variety of tracers are available that come with diverse costs (varying over an order of magnitude). Thus, the question is how to guide the selection for an ILE that does not risk being information-free while remaining economically efficient under high uncertainty about the intracellular fluxes. We applied the R-ED workflow (Figure 3) within the experimental setting described in section 2.3. The ARG and GLYC species are sampled in 10% steps resulting in 66 · 286 = 18,776 mixtures. Costs range from 10$ (unlabeled) to 25k$ (100% [1,3-^13^C_2_]-GLYC and 100% [U-^13^C_6_]-ARG). D-criterion values are related to a (hypothetical) reference ILE with a tracer mixture consisting of 100% [2-^13^C_1_]-GLYC and 100% [6-^13^C_1_]-ARG (16.6k$). The results of the R-ED workflow are collected in the aggregated data pool which is found in Supplementary Data S4.

#### 3.3.1. Exploration of the Metric Criteria Space

**Figure 4:**
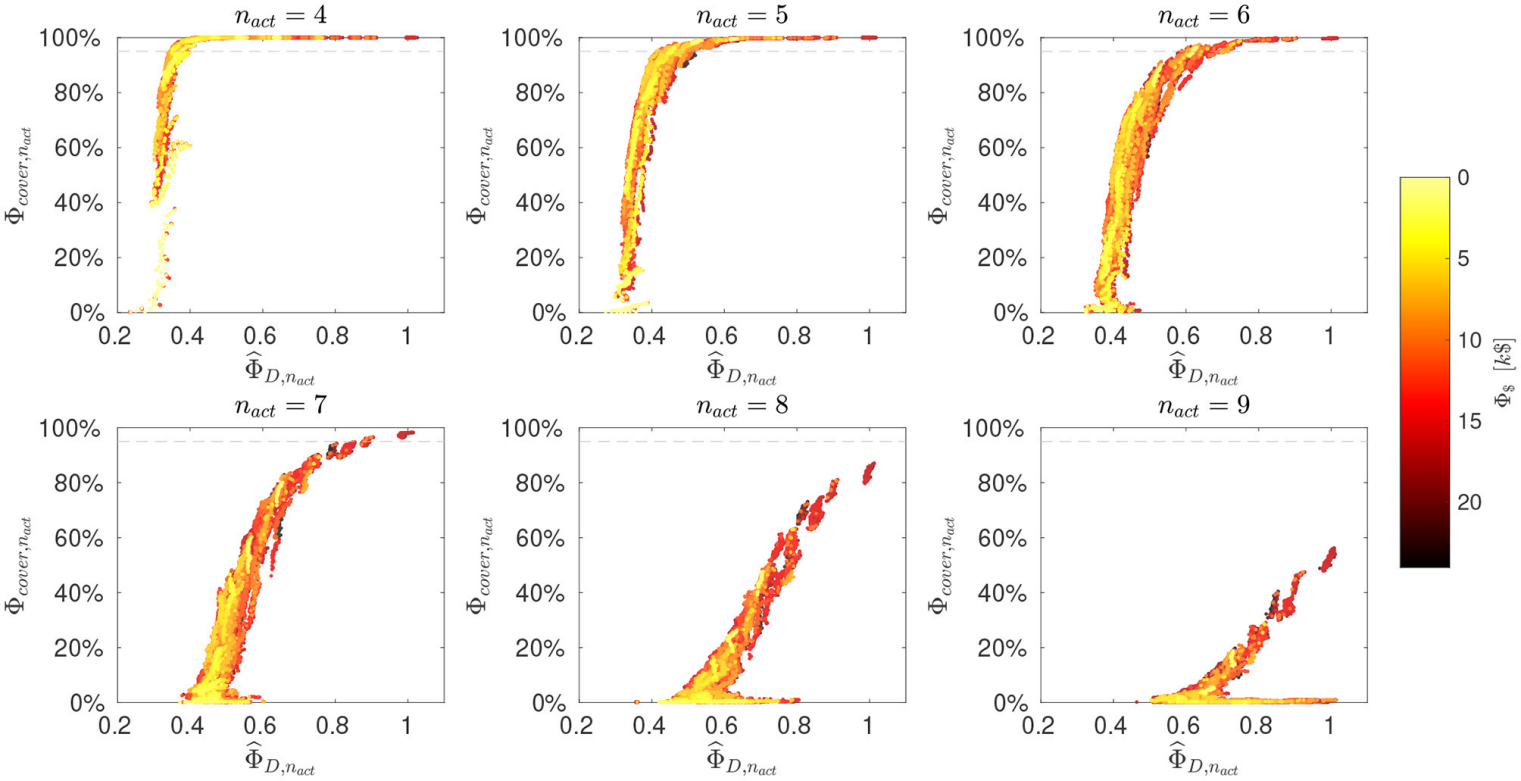
Evaluation of mixtures. Design criteria for selected *n*_*act*_ values (and for the remaining in Supplementary Information S1, Figure S1). Scatter plots of normalized D-criterion values (${\hat{\Phi }}_{D,n_{act}}$, larger values are better) versus coverage (Φ_*cover*_,_*n*_*act*__, larger values are better). The substrate costs (Φ_$_) are color-coded as indicated by the color bar. The horizontal dashed line indicates a Φ_*cover*_,_*n*_*act*__ value of 95%. Corresponding mixtures are available in the aggregate data pool (Supplementary Data S4).

Figure 4 summarizes the evaluation of mixtures as scatter plots, ordered according to the number of active fluxes (4 ≤ *n*_*act*_ ≤ 9). From the results the trade-off between flux identifiability and coverage becomes apparent. With larger *n*_*act*_, the coverage criterion decreases, meaning that it is less likely to find mixtures that assure a high number of identifiable fluxes over the whole possible flux space. The D- and coverage- criteria are highly non-linearly correlated. Nevertheless, for a fixed Φ_*cover*_,_*n*_*act*__, often a range of ${\hat{\Phi }}_{D,n_{act}}$ exists and vice-versa. In particular, mixtures can be found that are likely to identify a certain number of fluxes and do so well, or conversely, good choices for one flux sample are available that are often well-performing for many others, too. At the same time, there is the tendency that more informative (as measured with the D-criterion) designs are also more costly. Nonetheless, as we show below, there are often cheaper alternatives available that outperform more expensive mixtures.

#### 3.3.2. Choosing an Informative Mixture

For further evaluation, the aggregated data pool is consulted that contains all mixture characteristics computed by the R-ED workflow. The aggregated data pool is available in the Supplementary Data S4. Here, we summarize our main findings. With regard to tracer compositions, top performers contain high proportions of either [2-^13^C_1_]- or [1,3-^13^C_2_]-GLYC, where our evaluation shows that [2-^13^C_1_]-GLYC almost always outperforms the 50% more expensive [1,3-^13^C_2_]-GLYC. In contrast to GLYC, for ARG tracers no similarly clear picture emerges. Here, a broad range of ARG mixtures perform almost equally well. This gives flexibility in selecting the ARG mixture and allows making comparably cheap tracer choices.

For decision-making, as in the classical O-ED workflow, a value for *n*_*act*_ has to be chosen as a baseline for interpretation. Whereas in case of O-ED the choice has to be made a priori, in the R-ED workflow the decision is only made after inspecting the outcome, and it can be revised if considered appropriate. For our showcase, a reasonable choice is to select the highest *n*_*act*_ for which the coverage criterion is above 95%, as indicated by the dashed horizontal line in Figure 4, in our case *n*_*act*_ = 7.

For the further evaluation, we therefore fixed *n*_*act*_ to 7. Regarding the metrics, the highest coverage and D-criterion values are 98% and 1.01, respectively. They are obtained by a mixture of [2-^13^C_1_]-GLYC and 100% [U-^13^C_6_]-ARG with costs of 17.1k$ (Supplementary Information S1, Figure S2). This mixture is denoted *Mix 1* in the following. Interestingly, this is also one of the cheapest mixtures with comparable information quality. A common strategy in^13^C-MFA is to reduce labeling costs by “diluting” the tracer mixture with inexpensive naturally labeled substrates. Interrogation of the aggregated data pool shows that adding naturally labeled GLYC is of little utility in our case. Introducing 20% naturally labeled [^12^C]-GLYC reduces cost to only 13,9k$, but also lowers the D-criterion to 0.75 and the coverage to 88%. A by far better alternative is the mixture of 50% [^12^C]-, 40% [2-^13^C_1_]-, and 10% [1,3-^13^C_2_]-GLYC combined with 50% [^12^C]-, 40% [6-^13^C_1_]- and 10% [U-^13^C_6_]-ARG, termed *Mix 2*, which leads to cost of 9.2k$, a coverage of 96.5% and a D-criterion value of 0.90. This is also the cheapest mixture that satisfies a 95% coverage cut-off.

An often applied affordable substrate choice for ILEs with GLYC is to take a mixture of fully and naturally labeled GLYC (e.g., Beste et al., 2011; Alagesan et al., 2013). The best performing mixture of this type contains 10% fully labeled [U-^13^C_3_]-GLYC. The corresponding ARG mixture is e.g., 40% [U-^13^C_6_]- and 60% [^12^C]-ARG, henceforth denoted *Mix 3*. Consequently, the costs are comparatively low with 1.9k$, but the coverage and D-criteria are only moderate (80% and 0.66, respectively). Being among the cheapest mixtures, this composition is a good compromise for an efficient ILE. Figure S2 in Supplementary Information S1 shows the location of the selected mixtures in the context of the overall mixture evaluation.

While showing the exploratory strength of R-ED, the question remains how well a R-ED selected mixture performs compared to a mixture that is selected from a traditional O-ED. To benchmark our R-ED approach in this regard, we calculated the O-ED criterion value according to Equation (7) for the most likely flux map, i.e., the center of mass of the feasible flux space using the three GLYC species ([^12^C]-, [2-^13^C_1_]-, and [U-^13^C_3_]-GLYC), while fixing ARG to 100% [U-^13^C_6_]-ARG (Supplementary Table S3). The resulting mixture triangle is presented in Supplementary Information S1 (Figure S3). The most informative mixture is made up of 100% [2-^13^C_1_]-GLYC and 100% [U-^13^C_6_]-ARG, which is identical to *Mix 1*, in line with the results from our R-ED. The O-ED mixing triangle also shows an informative region with mixtures of 50% [U-^13^C_3_]- and 50% [^12^C]-GLYC (cost 8.8k$). A comparison with R-ED results reveals that this mixture provides a coverage of only 60%, and it is thus not very likely to identify 7 fluxes at least. Furthermore, the mixture is not very informative, with a normalized D-criterion value of only 0.6. This highlights the benefits of performing R-ED, as it shows that the results for one flux map are not representative for the whole flux space.

#### 3.3.3. Mixture Designs: An In-depth Look

**Figure 5:**
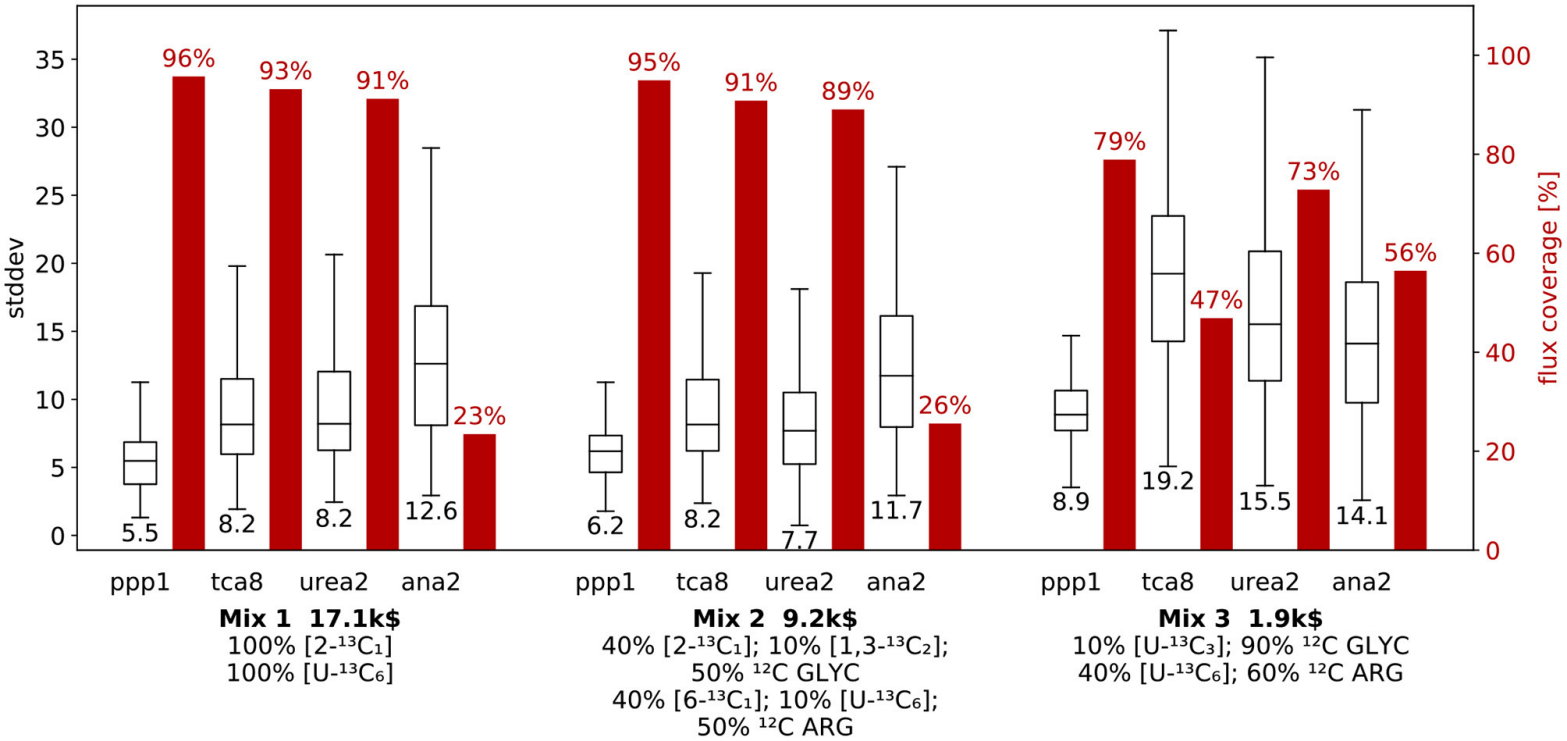
R-ED performance for three mixtures with respect to selected net fluxes. Comparison of information and cost criteria for mixtures *Mix 1, Mix 2*, and *Mix 3* with focus on the net flux through main pathways: pentose phosphate pathway (*ppp1*), tricarboxylic acid cycle (*tca8*), urea cycle (*urea2*), and the anaplerosis (*ana2*) (c.f. Figure 1). For each of the four fluxes, the box plots show the distribution of flux standard deviations over all flux samples, where the center line marks the median standard deviation (value given below the box plot). The red bars show the relative frequency with which the flux was found to be identifiable, i.e., the proportion of samples for which this particular flux was an active flux. Results for the remaining fluxes are given in Supplementary Information S1 (Figure S4).

With the three mixture candidates at hand, we next explore their performance in terms of specific flux information, i.e., dimension-reduced designs for *n*_*act*_ = 1. Thereby, we uncover specific weaknesses and strength of the mixtures, aiding the final design decision. For *Mix 1* to *3*, we exemplary investigate net fluxes of the main pathways: *ppp1* (pentose-phosphate pathway), *tca8* (tricarboxylic acid cycle), *urea2* (urea cycle), and *ana2* (anaplerosis) (cf. Figure 1). The results for these four fluxes are shown in Figure 5, and for the remaining fluxes in Supplementary Information S1 (Figure S4). For each of the mixtures, the proportion of flux samples, for which the particular flux was identified is given, along with the distribution of the corresponding flux standard deviations.

For each mixture, the flux standard deviations are qualitatively comparable, and each flux is identifiable with a standard deviation below 35 (referred to a GLYC uptake of 100). Among the fluxes shown, the *ppp1* flux is the best determinable flux for all three mixtures. Nonetheless, for the *ppp1, tca8* and *urea2* net fluxes the mixtures generally show a qualitatively very similar performance, where the coverage gradually drops from *Mix 1* to *3*, while the flux standard deviations are comparable for *Mix 1* and *Mix 2*, but increase for *Mix 3*. This effect is most prominent for *tca8*, where the coverage drops from 93% to 47%, while the median standard deviation increases from 8.2 to 19.2. In contrast, *Mix 3* is the best performing mixture for *ana2*, with a coverage of 56%, which means that it is more than twice as likely to identify *ana2* with *Mix 3* as it is with *Mix 1* or *Mix 2*. This discrepancy between the performance of the mixture for the fluxes stresses the importance of a flexible evaluation procedure, as a design that is considered favorable for all fluxes may indeed turn out to be sub-optimal for a specific set of fluxes.

Looking at the information metrics for the dimension-reduced designs (Figure 5 and Figure S2 in Supplementary Information S1), most of them (e.g., *ppp1, tca8, urea2*) show the same trends as the full design with *n*_*act*_ = 7. The full design represents an average over all fluxes. Nonetheless, some selected designs (for example *ana2*) exhibit a different characteristic than the average. Such deviations only become visible by taking a closer look at the information metrics.

## 4. Conclusion

Many existing O-ED methods for^13^C-MFA rely on information criteria that depend on the flux covariance matrix. Since the determination of this information matrix is based on first-order sensitivities, these methods may have poor performance when the underlying labeling system is a highly non-linear function of the fluxes. Furthermore, in the traditional O-ED workflow, proposed labeling strategies are commonly conditioned on an a priori choice of identifiable fluxes, which limits the exploration of potent tracer compositions. We here present the robustified tracer design workflow R-ED. R-ED enables the exploration of tracer mixtures that work well for all possible fluxes, rather than being confined to a single flux map. Besides the flux precision (confidence intervals) and the tracer costs, R-ED employs a new information metric, the so-called coverage, which characterizes the robustness of the tracer mixture in terms of flux identifiability. Instead of formulating R-ED as multi-objective problem, we opted for a sampling based approach, which allows us to explore the multi-variate design criteria landscape in-depth. Therewith, our work proposes a generalization of existing ED approaches, tailored to cases where the prediction of informative substrates has hitherto not been reliably possible.

We showcased the potential of R-ED by applying the workflow to the ILE design for *S. clavuligerus*, a potent antibiotic producer of CA, for which we identified highly informative, yet economic GLYC and ARG tracer mixtures. Particularly, we demonstrated the advantages of R-ED and its benefits over common *ad hoc* cost saving strategies such as the introduction of unlabeled substrates. Interestingly, as an example for highest informativeness, [2-^13^C_1_]-GLYC was preferred over the more expensive and often applied [1,3-GLYC_2_]. Application of R-ED to *S. clavuligerus* therefore enables the exploration of new labeling strategies to gain insights into central metabolic and production pathway fluxes in this organism. Of course, whether a designed labeling strategy is successful can only be revealed by conducting the ILE because any ED is only as good as its underlying assumptions. Thus, from the experimental perspective, implementing one of the proposed tracer mixtures to *S. clavuligerus* is the next step.

From the computational perspective, by its reliance on existing^13^C-MFA modules and by being independent of the network model, the R-ED workflow is generally applicable and easily transferable to other organisms for planing the very first ILE. In this context, we recommend the use of R-ED to minimize the risk of an information-less first experiment. By trading off design criteria in an exploration-based manner, users of R-ED can decide how to configure a cost-efficient ILE. After some knowledge about the fluxes has become available, this knowledge can be readily integrated as prior information into existing ED pipelines.

## Data Availability Statement

The original contributions presented in the study are included in the article/Supplementary Material, further inquiries can be directed to the corresponding author/s.

## Author Contributions

KN conceptualized and supervised the research. VP and MB set up the network model used in this work, implemented the computational workflow and performed computations. All authors analyzed the results, contributed to writing and revising the manuscript, and gave approval to the final version of the manuscript.

## Conflict of Interest

The authors declare that the research was conducted in the absence of any commercial or financial relationships that could be construed as a potential conflict of interest.

## Abbreviations

ARG: arginine
CA: clavulinic acid
ED: experimental design
GC-MS: gas chromatography-mass spectrometry
GLYC: glycerol
HDF5: hierarchical data format, version 5
ILE: isotope labeling experiment
MFA: metabolic flux analysis
O-ED: optimal experimental design
R-ED: robustified experimental design.
