# Supplementary material for: Robustifying Experimental Tracer Design for13C-Metabolic Flux Analysis
Source: Front Bioeng Biotechnol. 2021 Jun 22;9:685323. doi: 10.3389/fbioe.2021.685323 (PMC8258161; doi:10.3389/fbioe.2021.685323)
Supplement: Supplementary file 1 [file Data_Sheet_1.pdf]

Supplementary Material

Robustifying Experimental Tracer Design for ^{13}C-Metabolic Flux Analysis

Martin Beyß, Victor D. Parra-Peña, Howard D. Ramirez-Malule, Katharina Nöh

S1 TABLES AND FIGURES

Tracer	Product number	Price US$/g	Price kUS$ per 25g GLYC/0.2125g ARG
[U-$^{13}C_6$]-GLYC	CLM-1510-PK	526.00[a]	13.15
[1,3-$^{13}C_2$]-GLYC	CLM-1857-PK	839.00[a]	20.98
[2-$^{13}C_1$]-GLYC	CLM-1397-PK	540.00[a]	13.50
[^{12}C]-GLYC	G5516	0.36[b]	0.01
[U-$^{13}C_6$]-ARG	CLM-2265-H-PK	3449.00[a]	0.73
[6-$^{13}C_1$]-ARG	CLM-2070-PK	1446.00[a]	0.31
[^{12}C]-ARG	A5006	0.52[b]	0.00

Table S1. Isotopic tracer specification. Purity for all products is 99 atom%. The cost for a mixture is calculated by multiplying the relative abundances of GLYC and ARG tracers according to the required amounts (25g for GLYC and 0.2125g for ARG) with their respective costs.
[a] Cambridge Isotope Laboratories website as of August 24, 2020.
[b] Sigma Aldrich website as of August 24, 2020.

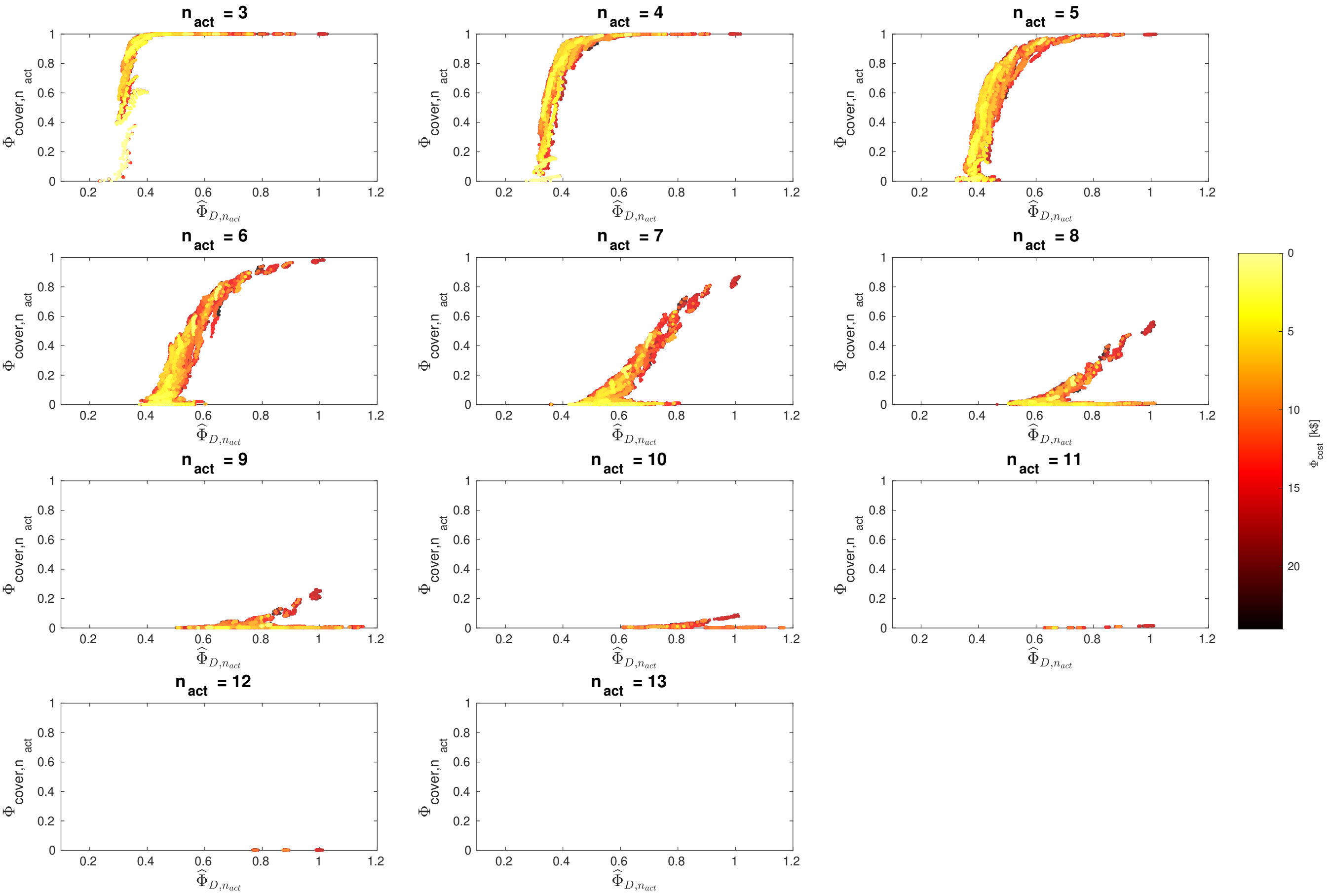

Figure S1. Evaluation of mixtures. Quality criteria for all n_{act} values. Scatter plots of normalized D-criterion values ($\widehat{\Phi}_{D,n_{act}}$) versus coverage ($\Phi_{cover,n_{act}}$). The substrate costs ($\Phi_{\$}$) are color coded as indicated by the color bar (yellow - low costs, dark red - high costs).

**Amino acid**	**Fragment (m/z)**	**Atoms (InChI)**	**Independent measurements**
Alanine	260	1,2	3
	232	1-3	2
Arginine	442	1-6	6
Aspartate	418 or 460	1-4	4
	316 or 390	1-3	3
	302	2,4	2
Glutamate	432 or 474	1-5	5
	330 or 404	1-4	4
	302	3,5	2
Glycine	246 or 288	1,2	2
	144 or 218	1	1
Histidine	440 or 482	1-6	6
	338 or 412	1-5	5
	302	5,6	2
Isoleucine	344	1-6	6
	200 or 274	1-5	5
Leucine	344	1-6	6
	200 or 274	1-5	5
Lysine	431 or 473	1-6	6
	329 or 403	1-5	5
	302	5,6	2
Methionine	320 or 362	1-5	5
	218 or 292	1-4	4
	302	4,5	2
Phenylalanine	336 or 378	1-9	9
	234 or 308	1-8	8
	302	8,9	2
Proline	286 or 328	1-5	5
	184 or 258	1-4	4
	302	4,5	2
Serine	390 or 432	1-3	3
	288 or 362	1,2	2
	302	2,3	2
Threonine	404 or 446	1-4	4
	376	1-3	3
Tyrosine	466 or 508	1-9	9
	364 or 438	1-8	8
	302	8,9	2
Valine	288 or 330	1-5	5
	186 or 260	1-4	4
	302	4,5	2
Total	41		167

Table S2. Labeling measurement specification. The configuration is composed from typical ^{13}C-MFA studies (Schmitz et al., 2017; Dauner and Sauer, 2000; Becker et al., 2008; Long and Antoniewicz, 2019). Atom numbers are given according to the IUPAC International Chemical Identifier (InChI, (Heller et al., 2013)).

Flux name	Flux constellation I	Flux constellation II
ana2.n	95.5	65.0
ca_out.n	29.0	6.6
co2_out.n	74.6	250.8
ppp1.n	97.1	110.8
tca8.n	-15.7	-27.3
urea2.n	33.4	81.1
nit1.n	40.0	40.0
ana3.x	59.0	29.8
emp3.x	15.4	1.2
emp4.x	26.4	55.0
emp5.x	40.3	7.2
emp6.x	0	0
nit2.x	77.3	70.0
nit8.x	17.6	58.3
ppp4.x	98.7	36.6
ppp5.x	23.3	95.2
ppp6.x	44.0	5.4
tca1.x	31.2	71.3
tca4.x	189.9	119.3
tca6.x	51.1	44.9
tca7.x	187.5	111.9
tca8.x	141.9	124.9

Table S3. Flux constellations. Independent flux values for the two flux constellations examined in Fig. 2 of the main text. .n – net flux, .x – exchange flux.

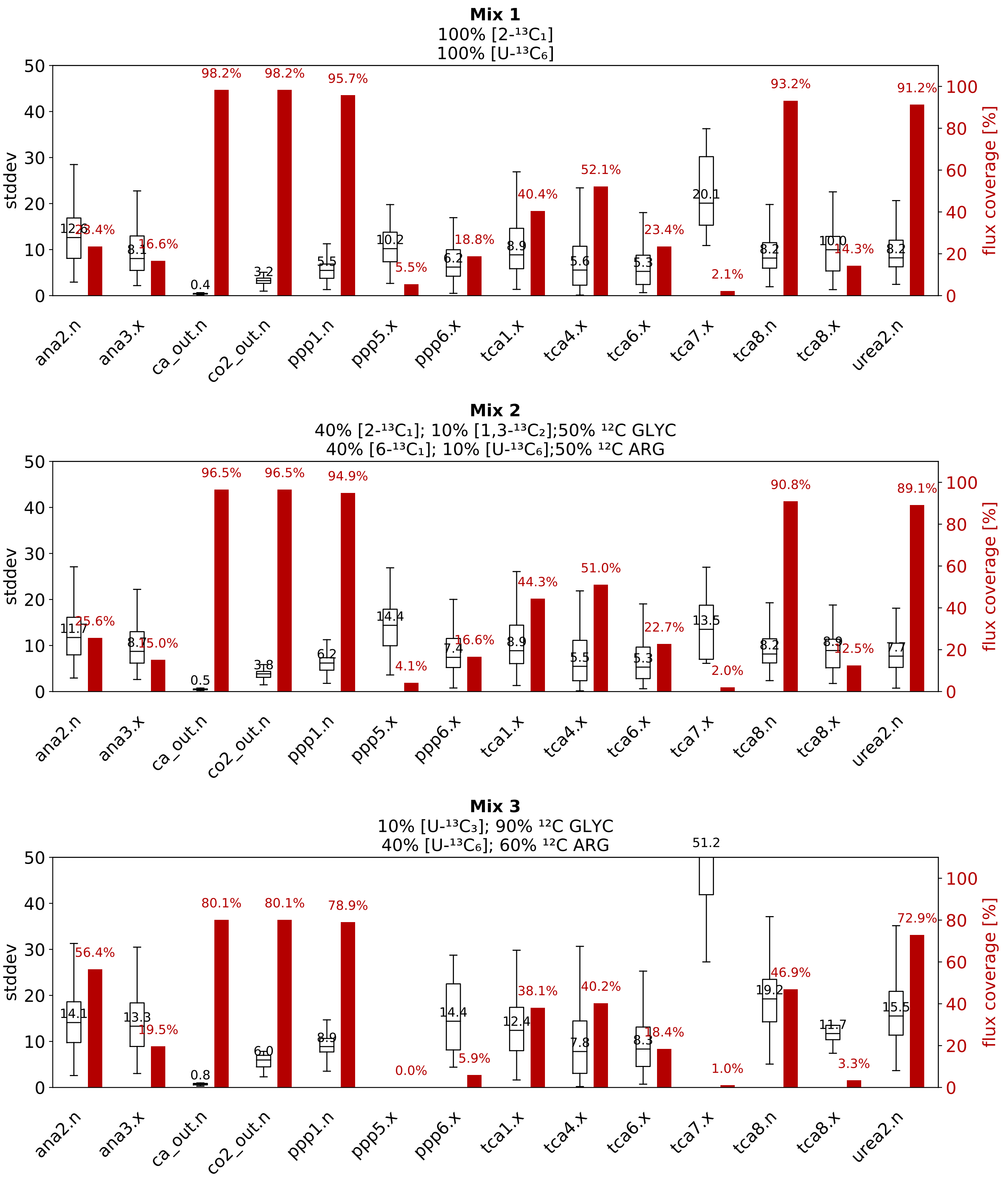

Figure S2. Comparison of metrics for three mixtures and the number of active fluxes $n_{act} = 7$**.** Per flux, the distribution of standard deviations of the flux samples is summarized as boxplot and the bar indicates the associated flux coverage (relative frequency with which the flux is found to be identifiable). .n – net flux, .x – exchange flux.

S2 COMPUTATION OF AGGREGATE DATA POOL

Here we describe how to set-up the R-ED workflow as depicted in Fig. 3 of the main text. The Supplementary Data S2 contains the FluxML model file `model.fml`, the mixture specification file `all.mix`, as well as the flux samples `samples.hdf5`. The flux samples are only required for the precise reproduction of the reported results, while executing the workflow with re-sampled fluxes will yield very similar, but independent results. We assume that all files are located in the current working directory of a Linux machine, that runs 13CFLUX2 (version 2.2 or later), the GNU parallel tools, python 3 and Matlab (R2019a or later, earlier versions may also work). Furthermore, the scripts `Global_Algorithm.py` and `Aggregate_all.m`, i.e. the contents of Supplementary Data S3, have to be present.

The sampling is done in two steps. First the actual sampling of the flux space is performed (512 samples) and the individual model files are created as shown in Listing S1. Notice, that when re-using existing samples, Line 2 has to be commented out. See documentation of 13CFLUX2 for further explanation of the `setfluxes` tool.

Listing S1. Sampling and transferring samples to model file

```
1 MODEL=model.fml
2 ssampler -i $MODEL -s nx -c all -n 512 -s nx -o samples.hdf5
3 mkdir -p models
4 for i in `seq -f '%03g' 512`;
5 do
6     setfluxes -i $MODEL -c all -l $i -H samples.hdf5 -o models/
          Model_sample_$i.fml
7 done
```

Afterwards, the `edscanner` tool is executed on the model set to compute the Jacobians for every mixture and every flux sample.

Listing S2. calculation of Jacobians

```
MIX=all.mix
mkdir -p models/edscan
for i in `seq -f '%03g' 512`;
do
    sem -j 64 "edscanner -i models/Model_sample_$i.fml -c all -m $MIX
        -o models/edscan/edscan_sample_$i.hdf5 -j -n 18876"
done
sem --wait
```

Subsequently, all Jacobians are processed to determine the active fluxes with the python script `Global_Algorithm.py`. The final aggregation is achieved by the script `Aggregate_all.m`, which is executed within Matlab. Finally the aggregated data pool is exported into a table, which can also be found in the Supplementary Data S4.

REFERENCES

Becker, J., Klopprogge, C., and Wittmann, C. (2008). Metabolic responses to pyruvate kinase deletion in lysine producing *Corynebacterium glutamicum*. *Microbial Cell Factories* 7, 8. doi:10.1186/1475-2859-7-8

Dauner, M. and Sauer, U. (2000). GC-MS analysis of amino acids rapidly provides rich information for isotopomer balancing. *Biotechnology Progress* 16, 642–649. doi:10.1021/bp000058h

Heller, S., McNaught, A., Stein, S., Tchekhovskoi, D., and Pletnev, I. (2013). InChI - The worldwide chemical structure identifier standard. *Journal of Cheminformatics* 5, 7. doi:10.1186/1758-2946-5-7

Long, C. P. and Antoniewicz, M. R. (2019). High-resolution ^{13}C metabolic flux analysis. *Nature Protocols* 14, 2856–2877. doi:10.1038/s41596-019-0204-0

Schmitz, A., Ebert, B. E., and Blank, L. M. (2017). *GC-MS-based determination of mass isotopomer distributions for ^{13}C-based metabolic flux analysis* (Berlin, Heidelberg: Springer), chap. 13. 223–243. doi:10.1007/8623_2015_78
